# Dynamic Network-Based Relevance Score Reveals Essential Proteins and Functional Modules in Directed Differentiation

**DOI:** 10.1155/2015/792843

**Published:** 2015-04-21

**Authors:** Chia-Chou Wu, Che Lin, Bor-Sen Chen

**Affiliations:** ^1^Control and Systems Biology Laboratory, National Tsing Hua University, Hsinchu 30013, Taiwan; ^2^Institute of Communication, National Tsing Hua University, Hsinchu 30013, Taiwan; ^3^Department of Electrical Engineering, National Tsing Hua University, Hsinchu 30013, Taiwan

## Abstract

The induction of stem cells toward a desired differentiation direction is required for the advancement of stem cell-based therapies. Despite successful demonstrations of the control of differentiation direction, the effective use of stem cell-based therapies suffers from a lack of systematic knowledge regarding the mechanisms underlying directed differentiation. Using dynamic modeling and the temporal microarray data of three differentiation stages, three dynamic protein-protein interaction networks were constructed. The interaction difference networks derived from the constructed networks systematically delineated the evolution of interaction variations and the underlying mechanisms. A proposed relevance score identified the essential components in the directed differentiation. Inspection of well-known proteins and functional modules in the directed differentiation showed the plausibility of the proposed relevance score, with the higher scores of several proteins and function modules indicating their essential roles in the directed differentiation. During the differentiation process, the proteins and functional modules with higher relevance scores also became more specific to the neuronal identity. Ultimately, the essential components revealed by the relevance scores may play a role in controlling the direction of differentiation. In addition, these components may serve as a starting point for understanding the systematic mechanisms of directed differentiation and for increasing the efficiency of stem cell-based therapies.

## 1. Background

Stem cell-based therapies require precise control of the processes involved in the differentiation of pluripotent stem cells into specific cell types. For therapeutic purposes, the induced stem cells have to proliferate and differentiate to produce a sufficient quantity of the desired cell type; however, proliferation and differentiation have to be controlled to prevent abnormal proliferation (e.g., a source of cancer). Embryogenesis exemplifies the concept of balance between promotion and restriction of the proliferation and differentiation of stem cells. Several experimental protocols to induce stem cells toward a desired differentiation direction have therefore been developed based on knowledge of the molecular mechanisms of embryogenesis. Most of these protocols successfully produce the desired outcomes by manipulating the regulators or members of embryogenesis-related pathways.

For example, in the directed differentiation of human pluripotent cells into ureteric bud kidney progenitor-like cells, a new platform for renal lineage commitment was developed by adding the required molecules during kidney embryogenesis [1]. A robust and efficient process to direct differentiation using a series of temporal growth factor manipulations that mimicked embryonic intestinal development was established for the directed differentiation of human pluripotent cells into intestinal tissue [2]. In the dual inhibition of SMAD signaling, a possible mechanism was presented and a highly efficient neural conversion was performed [3]. A key objective for neural induction in human embryonic stem cells (hESCs) is the knowledge and capability to initiate signaling pathways [4]. The derivation of floor plate (FP) precursors was induced by a modified dual-SMAD inhibition protocol, progressing to dopamine (DA) neurons with fibroblast growth factors (FGF) and sonic hedgehog (SHH) signals that have the ability to control dopaminergic cell production. Under both LSB and LSB/S/F8 control conditions, and based on previous studies of dual-SMAD inhibition of neural induction, a new midbrain DA neuron protocol was developed [5]. Exposure to CHIR99021 (CHIR), a potent GSK3B inhibitor, was found to activate WNT signaling [6]. Protocols that aim at known or specific pathways and mechanisms to control differentiation work on the basis of the assumption that if these mechanisms are invoked, then the desired cell types can be obtained. Despite the successful examples mentioned above, we believe that there are still numerous failures in the design of effective protocols to control the differentiation outcomes. These failures can be attributed to a lack of knowledge about the systematic mechanisms of directed differentiation.

The directed differentiation process from stem cells to specific neuron types through the induction of a series of molecular events in a systematic manner has proven difficult because the normal developmental process that generates most classes of CNS neurons remains poorly defined [7]. However, in the directed differentiation of mouse induced pluripotent stem cells (iPSCs) into cardiovascular cells, researchers succeeded in inducing the correct direction of differentiation based on systematic knowledge of the differentiation process of cardiovascular cells from iPSCs [8]. Thus, systematic understanding of the mechanisms of directed differentiation can provide a systematic perspective for designing protocols. The molecular interaction network of differentiation can provide promising molecular or modular candidates to balance the promotion and restriction of stem cell growth and to control the differentiation direction.

In a modified dual-SMAD protocol study [5], the authors induced three different outcomes using three different treatment schemes (LSB, LSB/S/F8, and LSB/S/F8/CHIR), then measured the temporal gene expression profiles for each treatment, and verified that the derived DA neurons were engraftable and had the same activity as endogenous DA neurons. The three outcomes are regarded as three stages in the directed differentiation of iPSCs to DA neurons. According to the temporal gene expression profiles of the three treatments, we constructed protein-protein interaction networks for the three stages in the differentiation process. The constructed networks describe the dynamics of the proteins as the interactions between proteins. The dynamic network modeling of biological processes has been acknowledged as furthering our understanding of these processes from systematic viewpoints [9, 10]. Through a comparison of the three networks and the phenotypes observed under the three treatment schemes, we utilized the interaction difference networks to identify proteins with large interaction activity variation. As protein-protein interactions (PPIs) are essential elements in biological processes, the roles of the most variable components of the PPIs in directed differentiation are worth further examination. We propose a relevance score to quantify the importance of proteins and functional modules in the directed differentiation process. Based on these scores, we discuss how the balance between promotion and restriction of stem cell proliferation and differentiation is achieved and how the differentiation direction is controlled. We believe that this may serve as a starting point for understanding the systematic mechanisms of directed differentiation and increase the efficiency of stem cell-based therapies.

## 2. Methods

In this study, the analysis procedure used to identify essential proteins and functional modules in the directed differentiation process consists of three key steps: (i) microarray data preprocessing, (ii) dynamic PPI network construction, and (iii) relevance score calculation from IDNs between the different differentiation stages. Details for each step are described in the following sections.

### 2.1. Microarray Data Preprocessing

**Figure 1:**
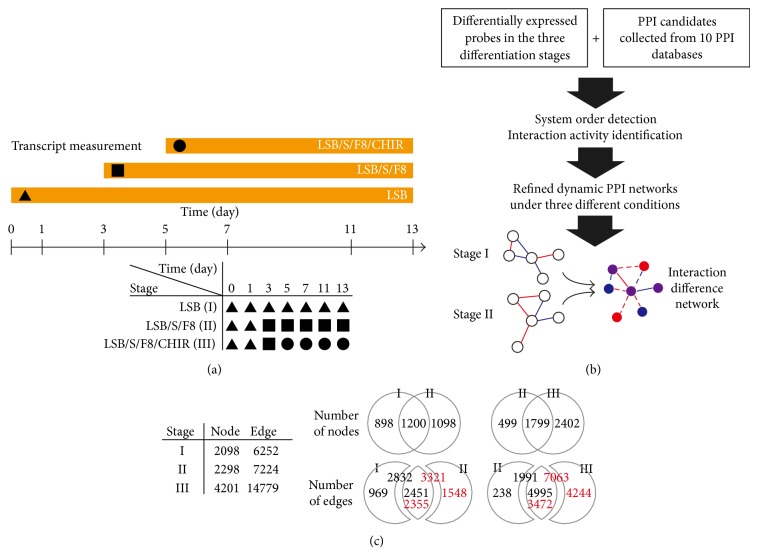
Overview of data, flowchart, and summary of the dynamic PPI networks in the differentiation process from hESCs to DA neurons. (a) The upper part of the timeline shows three experimental conditions (LSB: ▲, LSB/S/F8: ■, and LSB/S/F8/CHIR: ●) used in [5]. The numbers on the timeline indicates the sampling time points of microarray data (days 0, 1, 3, 5, 7, 11, and 13). The lower part of the timeline shows the three differentiation stages comprising of the data from the three experimental conditions. (b) A brief flowchart of dynamic network construction. Using PPI candidates from databases, the data of the three differentiation stages, and dynamic network modeling, three PPI networks corresponding to three differentiation stages are constructed. Then the interaction difference network (IDN) can be derived from the two networks of differentiation stages. (c) In the table at left, it shows the total number of nodes and edges at three stages. The Venn diagram at top-right shows the distribution of number of nodes between stage I and stage II and between stage II and stage III. The Venn diagram at lower right shows the distribution of number of edges between stage I (black) and stage II (red) and between stage II (black) and stage III (red).

The genome-wide microarray data used in this study were obtained from the GSE32658 dataset in the GEO database [5] using an Expression BeadChip Kit (Illumina, San Diego, CA, USA). The data consist of measurements of temporal gene expression in the directed differentiation of hESCs into DA neurons over a range of 0 to 13 days under three different differentiation conditions (LSB, LSB/S/F8, and LSB/S/F8/CHIR) (Figure 1(a)). For each condition, there are three replications for each time point (days 0, 1, 3, 5, 7, 11, and 13) used in this study. The microarray data were obtained from a study conducted by Kriks et al. According to GEO database documentation, no normalization or background correction was performed on the GSE32658 dataset. We therefore performed simple quantile normalization and selected differentially expressed genes using MATLAB for data preprocessing before network construction. We defined three consecutive differentiation stages over 13 days according to the three induction conditions in [5] and the resultant phenotypes of these induction conditions. The first stage corresponding to the LSB induction condition (i.e., the iPSCs were only induced by LSB at the beginning of the experiments) consisted of the temporal gene expression profiles at 0, 1, 3, 5, 7, 11, and 13 days under the LSB induction condition. The second stage corresponding to the LSB/S/F8 induction condition (i.e., the iPSCs were induced by LSB at the beginning of the experiments and then SHH and FGF8 at one day after LSB addition) consisted of the temporal gene expression profiles at 0 and 1 days under the LSB induction condition and 3, 5, 7, 11, and 13 days under the LSB/S/F8 induction condition. The third stage corresponding to the LSB/S/F8/CHIR induction condition (i.e., the iPSCs were first induced by LSB at the beginning of the experiments, SHH and FGF at one day after LSB addition, and CHIR at three days after LSB addition) consisted of the temporal gene expression profiles at 0 and 1 days under the LSB induction condition, 3 days under the LSB/S/F8 induction condition, and 5, 7, 11, and 13 days under the LSB/S/F8/CHIR induction condition. The resultant phenotypes of the three stages are dorsal forebrain precursors, ventral/hypothalamic precursors, and midbrain DA neurons, respectively (Figure 1(a)). Data were normalized relative to the controls to avoid masking biological effects [11]. To construct the PPI network, differentially expressed probes were further filtered from the normalized data using an ANOVA and a significance threshold of FDR-corrected *P* values < 1 × 10^−3^. After filtering, there were 3556, 3731, and 6460 differentially expressed genes for the three differentiation stages, respectively, which were used to investigate and construct the dynamic PPI networks.

### 2.2. Dynamic PPI Network Construction

The flowchart for the dynamic PPI network construction used in this study is summarized in Figure 1(b) and briefly described in the following text.

First, the candidate PPIs from different databases were collected before identifying the interaction activities between the differentially expressed proteins in the dynamic PPI network model. These PPI candidates consisted of interactions from computational predictions and biological experiments. We collected candidates from 10 frequently used PPI databases (BIND [12], BioGrid [13], DIP [14], HPRD [15], I2D [16], INTACT [17], MINT [18], PIP [19], Reactome [20], and STRING [21]). The candidate PPIs were then pruned using the microarray data to construct the realistic dynamic PPI network.

The logic of our methodology to construct a network is that the experimental data (e.g., the microarray in this study) are used to examine all potential protein-protein interactions. We then considered the PPI information from the databases as the potential PPIs rather than all possible interactions, due to the computation complexity. Therefore, if PPI information was not included in the database, it does not appear in our constructed networks. Moreover, the sources of PPI information, such as text mining or interlogue mapping, reflect the confidence in the reality of the PPIs. Hence, we pooled all PPIs from the different sources to avoid missing PPI information if a specific source was used as a criterion to construct a network. However, this also increases the number of false positive PPIs if PPIs of different sources are integrated. This was addressed by using the experimental data to examine possible PPIs to reduce bias.

A discrete dynamic PPI model was employed to describe the PPIs in the network at different differentiation stages. The dynamic model for a target protein *p* in the candidate PPI network was proposed as follows [22]:

(1) zpt+1=zp[t]+∑q=1Qpbpqzp[t]zq[t]+αpxp[t] −βpzpt+ωpt+1,

where *z*_*p*_[*t*] is the protein level of *p* at time *t*, *b*_*pq*_ is the interaction activity of the *q*th protein and the *p*th target protein, *z*_*q*_[*t*] is the protein level of the *q*th protein that interacts with the target protein *p* at time *t*, *α*_*p*_ is the translation effect from mRNA to protein, *x*_*p*_[*t*] is the corresponding mRNA expression level of the target protein *p*, *β*_*p*_ is the degradation rate of the target protein *p*, and *ω*_*p*_[*t*] is stochastic noise. The biological interpretation of (1) is that the protein level of the target protein *p* at time *t* + 1 is associated with the current target protein level, the regulatory interactions with *Q*_*p*_ interactive proteins, the translation effect from mRNA of target protein *p*, the degradation effect, and stochastic noise at time *t* + 1. As the protein-protein interaction is in fact a chemical reaction of two proteins, we adopted the formulation of the rate equation for a chemical reaction, that is, the rate coefficient multiplied by the product of the reactants. Dynamic PPI modelling of *p* in (1) can be represented using the following regression form:

(2) zpt+1=zptz1t⋯zptzQtxptzpt ·bp1⋮bpQαp1−βp+ωpt+1≜ϕpt ·θp+ωpt+1,

where *ϕ*_*p*_[*t*] is the regression data vector and *θ*_*p*_ is the kinetic parameter vector to be identified. In order to avoid overfitting to a small amount of data when identifying parameters, original data points were interpolated to *L* data points using a cubic spline method [23, 24]. The desirable data size number, *L*, is approximately 3–5 times the number of estimated parameters. Thus, after assembling the *L* data points, (2) can be rewritten as the following equation:

(3) $$\begin{array}{c} Z_{p}=\Phi_{p}\cdot \theta_{p}+\Omega_{p}, \end{array}$$

where *Z*_*p*_ = [*z*_*p*_[1],…,*z*_*p*_[*L*]]^*T*^, Φ_*p*_ = [*ϕ*_*p*_[0],…,*ϕ*_*p*_[*L* − 1]]^*T*^, and *Ω*_*p*_ = [*ω*_*p*_[1],…,*ω*_*p*_[*L*]]^*T*^. The parameter identification problem can then be formulated as the following least square minimization problem:

(4) $$\begin{array}{c} \underset{\theta_{p}}{\min}\frac{1}{2}{\left\|Z_{p}-\Phi_{p}\cdot \theta_{p}\right\|}_{2}^{2}. \end{array}$$

Because large-scale measurements of protein levels are still lacking, mRNA expression profiles were used as substitutes for protein levels when identifying the protein interaction parameters. Even if the mRNA expression level is not completely representative of the protein expression level, there are partly positive correlations between them [25, 26]. Therefore, the mRNA microarray can represent the trend of the corresponding protein expression level; that is, we assume that the gene expression level is representative of the protein expression level. Once the protein interaction activity *b*_*pq*_ was estimated, Akaike's information criterion (AIC) [27, 28] was applied to eliminate models with false positive protein interactions. This model order detection technique has been widely used in different fields (e.g., ecology, signal processing, engineering, systems biology, etc.) and can provide a fair view of the elimination of false positive interactions based on the microarray data when compared with the curated information. Realistic PPI networks were then constructed from the remaining candidate PPI networks. Details of the dynamic network construction can be found in [22].

### 2.3. Relevance Score Calculation from Interaction Difference Networks (IDNs) under Different Differentiation Stages

The resultant PPI networks at different differentiation stages can be represented as a matrix according to the constructed dynamic PPI networks:

(5) $$\begin{array}{c} \left[\begin{array}{ccc} {\hat{b}}_{11}^{s} & \cdots & {\hat{b}}_{1Q}^{s}\\ \vdots & \ddots & \vdots \\ {\hat{b}}_{Q1}^{s} & \cdots & {\hat{b}}_{QQ}^{s} \end{array}\right],\;s\in \left\{\text{LSB},\text{LSB}/\text{S}/\text{F}8,\text{LSB}/\text{S}/\text{F}8/\text{CHIR}\right\}, \end{array}$$

where ${\hat{b}}_{pq}$ is the interaction activity between protein *p* and protein *q* in the realistic PPI networks and the superscript *s* of interaction activity ${\hat{b}}_{pq}$ represents the differentiation stage. The interaction difference of two dynamic PPI networks at different differentiation stages is called the interaction difference network (IDN) in the differentiation process of hESCs into DA neurons and can be expressed as the following interaction difference matrix:

(6) Ds2−s1=d11⋯d1Q⋮⋱⋮dQ1⋯dQQ=b^11s2⋯b^1Qs2⋮⋱⋮b^Q1s2⋯b^QQs2 −b^11s1⋯b^1Qs1⋮⋱⋮b^Q1s1⋯b^QQs1s1, s2∈LSB,LSB/S/F8,LSB/S/F8/CHIR,

where *d*_*pq*_ is the difference in the interaction activity of protein *p* and protein *q* in the IDN between two different differentiation stages. For essential proteins in the directed differentiation process, two issues are taken into consideration. First, the magnitude of the interaction activity represents the influence of one protein on the other proteins in the realistic PPI network. Second, if a protein plays a critical role in the transition from differentiation stage 1 to differentiation stage 2, the difference in the interaction activities will be larger. Based on these considerations, the relevance score (RS) of a protein in the directed differentiation process can be defined as follows:

(7) $$\begin{array}{c} RS_{p}=\frac{\sum_{q=1}^{Q_{p}}\left|d_{pq}\right|}{\text{degree}\;\text{of}\;\text{protein}\;p}, \end{array}$$

where degree of protein *p* is the number of nonzero elements in the *p*th row of the interaction difference matrix *D*. Similarly the relevance score of the functional module *f*, a set of proteins with a specific function, in the directed differentiation process can also be defined:

(8) $$\begin{array}{c} RS_{f}=\underset{p\in f}{\sum }\frac{\sum_{q=1}^{Q_{p}}\left|d_{pq}\right|}{\text{degree}\;\text{of}\;\text{protein}\;p}. \end{array}$$

Classification of a group of proteins in the IDNs into functional modules or pathways can easily be achieved by ontological analyses, and relevance scores of the functional modules can be calculated accordingly. Similarly, the relevance score of the functional module can quantify the importance of a functional module in the directed differentiation.

## 3. Results and Discussion

### 3.1. Dynamic Networks and Interaction Variations of Well-Known Proteins at Three Differentiation Stages

**Figure 2:**
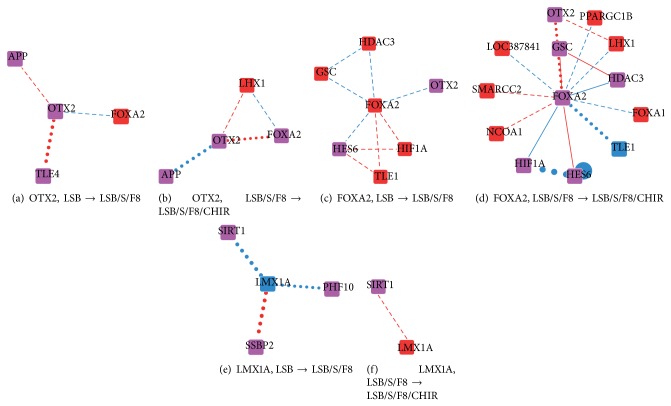
The interaction activity variations of well-known proteins OTX2, FOXA2, and LMX1A in the IDNs at two differentiation stage transitions. (a) Interaction activity variation of OTX2 from LSB to LSB/S/F8; (b) of OTX2 from LSB/S/F8 to LSB/S/F8/CHIR; (c) of FOXA2 from LSB to LSB/S/F8; (d) of FOXA2 from LSB/S/F8 to LSB/S/F8/CHIR; (e) of LMX1A from LSB to LSB/S/F8; (f) of LMX1A from LSB/S/F8 to LSB/S/F8/CHIR. (Nodes: red indicates disappearance at the previous stage and appearance at the later stage. Blue indicates appearance at the previous stage and disappearance at the later stage. Purple indicates appearance under both conditions. Edges: red indicates an enhancing interaction (i.e., + → ++, − − → −, 0 → +, and − → 0). Blue indicates an attenuating interaction (++ → +, − → − −, + → 0, and 0 → −). A solid edge represents appearance under both conditions. A dashed edge represents disappearance under the previous condition and appearance under the later condition. A dotted edge represents appearance under the previous condition and disappearance under the later condition. The width of the edge represents the magnitude of the interaction activity variations.)

In [5], Kriks et al. indicated that the failure of past strategies to induce the derivation of DA neurons is due to incomplete specification. These authors presented a novel strategy to complete the specification of DA neurons: a scalable source for neural transplantation. Three induction conditions (LSB, LSB/S/F8, and LSB/S/F8/CHIR) were used to clarify the required elements for DA neuron specification. The LSB induction condition yielded dorsal forebrain neuron identity; LSB/S/F8 yielded ventral/hypothalamic neuron identity; and LSB/S/F8/CHIR yielded midbrain DA neuron identity. We therefore defined the three differentiation stages of pluripotent stem cells (PSCs) into dopamine (DA) neurons corresponding to the three induction conditions. From this a natural and basic question concerning the parts of the induced PSCs (which can be viewed as dynamic and PPI networks) follows that are varied concurrent with the evolution of the three stages, as these varied parts may play crucial roles in influencing the differentiation direction. Dynamic network modeling techniques were therefore used to construct dynamic PPI networks to quantitatively describe the highly dynamic and complex PPIs of the three differentiation stages. The resultant dynamic PPI networks are summarized in Figure 1(c). To investigate the variations in the transition between two differentiation stages, the interaction difference matrices between stages (*D*_LSB/S/F8-LSB_ and *D*_LSB/S/F8/CHIR-LSB/S/F8_) were calculated following (6), allowing the visualization of the IDNs (see File S1 in Supplementary Material available online at http://dx.doi.org/10.1155/2015/792843 for all IDNs). Furthermore, a relevance score proposed to quantify the variation of interaction activity of proteins between stages was calculated from the interaction difference matrices. Prior to further analysis of the IDNs in the directed differentiation process, we assessed the plausibility of relevance scores by examining the scores of well-known key proteins [5] (OTX2, FOXA2, and LMX1A) for DA neuron differentiation from hESCs and their first neighbors in the IDNs (see Figure 2).

OTX2, the first transcription factor proven to have a role in mesencephalic-diencephalic dopaminergic (mdDA) neurogenesis [29], controls the development of several neuronal populations in the midbrain by regulating progenitor identity and neurogenesis [30]. After adding SHH and FGF8, the positive interaction between OTX2 and APP and the negative interaction between OTX2 and FOXA2 emerged, and the negative interaction between OTX2 and TLE4 diminished (Figure 2(a)). Because TLE4 negatively regulates the transactivating ability of OTX2 [31], reducing the negative effect of TLE4 on OTX2 may enhance the ability of OTX2 to determine cell fate during directed differentiation. After adding CHIR, the positive interaction between OTX2 and APP and the negative interaction between OTX2 and FOXA2 diminished, and the positive interaction between OTX2 and LHX1 and the negative interaction between LHX1 and FOXA2 emerged (Figure 2(b)). The emerging positive interaction between OTX2 and APP and its following disappearance reflect the fact that overexpression of the APP gene causes glial differentiation of stem cells and that reduction in APP levels would be useful in promoting neurogenesis [32]. This indicates the necessity of CHIR addition to promote adequate neural differentiation. The connection between OTX2 and FOXA2 is more intricate. After adding SHH and FGF8, a negative interaction emerged; the addition of CHIR then turned the direct negative interaction into indirect interactions through LHX1, which may promote DA differentiation [33].

FOXA2, a transcriptional activator, regulates DA neuronal generation and differentiation [34] and is only significantly differentially expressed after adding SHH and FGF8 (Figure 2(c)). The negative interaction between FOXA2 and HES6 emerged after adding SHH and FGF8, and FOXA2 exerted indirect positive interactions on HES6 through HIF1A and TLE1. Later, the negative interaction was attenuated by adding CHIR and the indirect positive interactions were diminished (Figure 2(d)). HES6, a candidate gene for mood disorder susceptibility and antidepressant response, promotes neuronal differentiation [35]. Thus, the interactions between FOXA2 and HES6 may provide a mechanism to control the differentiation directly and indirectly. The positive interaction between FOXA2 and SMARCC2 (also known as BAF170) emerged after adding CHIR. SMARCC2 has been proven to play a role in neurogenesis [36]. The negative interaction between FOXA2 and PPARGC1B (also known as PGC1B), which controls mitochondrial metabolism [37], emerged after adding CHIR. The positive interaction between FOXA2 and NCOA1 (also known as SRC1) emerged after adding CHIR. SRC-1 null mice show moderate motor dysfunction and delayed development of cerebellar Purkinje cells [38].

LMX1A, a roof plate marker, indicates the establishment of midbrain DA neuron precursor fate [5] and was diminished after SHH and FGF addition but emerged again after CHIR addition (Figures 2(e) and 2(f)). The interactions between LMX1A and SIRT1 declined throughout the differentiation process. SIRT1 mediates MPP+-induced apoptosis in dopaminergic cells [39]. This may imply that control of apoptosis is exerted at the beginning of the differentiation process and is progressively attenuated.

The interaction variations between these three key proteins and their first neighbors provide insights into the mechanisms of how these proteins determine the direction of differentiation. The phosphorylation events are indeed key events of information transmission in the signaling pathways. The results of signaling pathways are related to the activation or inhibition of gene expressions, which are in turn used to estimate interaction strengths in the network. We may therefore state that the effects of phosphorylation are implicit in the interaction strengths of the constructed networks. Although their neighbors have been studied to some extent in literature, here the IDNs of the differentiation stage transitions linked them and are related to the three key proteins. In addition to the qualitative descriptions of the interaction variations, we also calculated the relevance scores for each protein in the IDNs. The width of the links of the three key proteins delineated their high relevance scores. Thus, we use the relevance scores in the following sections to evaluate the importance of proteins with higher scores and investigate their roles in directed differentiation.

### 3.2. Proteins with the Top Ten Relevance Scores in the Interaction Difference Networks

**Figure 3:**
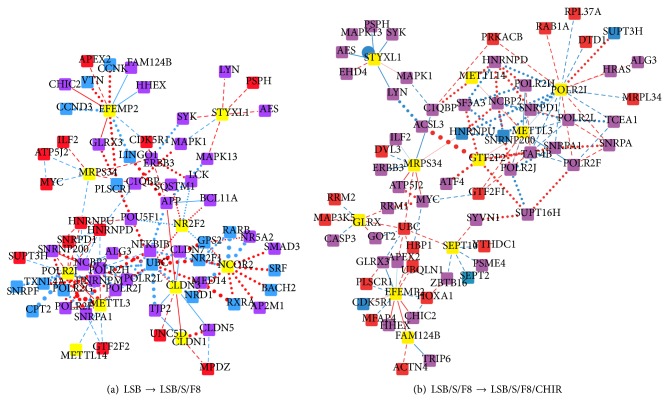
Interaction activity variations of the ten proteins with the highest relevance scores in the IDNs at two differentiation stage transitions. (a) Interaction activity variations of the ten proteins highest in relevance score from LSB to LSB/S/F8. (b) Interaction activity variations of the ten proteins highest in relevance score from LSB/S/F8 to LSB/S/F8/CHIR. Node and edge colors and edge line styles have the same interpretation as in Figure 2. A yellow node represents a protein with a relatively high relevance score (Table 1).

Following the analysis of the three key proteins of the directed differentiation, we here provide quantitative measurements of the interaction activity variations of the proteins in the transitions of the differentiation stages according to the IDNs. The data record three different differentiation processes from the same hESCs into three different cell types based on three different protocols. Only one protocol has the potential to develop engraftable DA neurons for further medical treatments of Parkinson's disease. The other two protocols resulted in some unexpected or unwanted cell types, which may cause improper cell growth. Our primary goal was to determine why the protocol can result in the development of DA neurons. Interactions that differ from those described in the “LSB/S/F8/CHIR” protocol are therefore considered as potential causes for unexpected or unwanted results. A similar evaluation based on IDNs has provided insight into the pathways and mechanisms of lung carcinogenesis and potential therapeutic targets for lung cancer [40]. Utilizing the identified interaction matrices (5) from microarray data and the definition of the relevance score of a protein in (7), the importance of a protein at different stages of the directed differentiation process of hESCs to DA neurons can be evaluated. The proteins with the top ten relevance scores are shown in Table 1 and the subnetwork comprising these proteins extracted from the whole IDN is shown in Figure 3.

The METTL3-METTL14 complex showed a negative interaction after adding SHH and FGF8. This complex, a methyltransferase, mediates mammalian nuclear RNA N^6^-adenosine methylation [41], which is essential for neural stem cell differentiation [42]. In both of the IDNs (*D*_LSB/S/F8-LSB_ and *D*_LSB/S/F8/CHIR-LSB/S/F8_), this complex had a strong interaction with POLR2I, a transcription factor related to Huntington's disease. Although the role of POLR2I in the directed differentiation of hESCs into DA neurons remains unclear, the strong interaction may imply the importance of methylation and POLR2I in the differentiation process. Furthermore, the mitochondrial ribosomal protein MRPS34 interacted with a human homolog of the* Drosophila* discs-large tumor suppressor protein (hDLG), which may function in the regulation of development and cell death [43]. The differential expression of MRPS34 was also reported in several stem cell lines. EFEMP2 and STYXL1 are related to EGF and the cell cycle, respectively, and their roles in directed differentiation are still not fully explored. The above proteins are common in both IDNs, indicating that methylation, mitochondrial ribosomes, EGF signaling, and cell cycle-related functions are all involved in differentiation stage transitions.

In the IDN between LSB/S/F8 and LSB conditions (*D*_LSB/S/F8-LSB_; Figure 3(a)), the ligand-activated transcription factor NR2F2 is involved with OCT4 in mammalian ESC pluripotency [44] and FOXA1 transcription factor networks [45]. Its interaction activity with NCOR2, a nuclear receptor co-repressor controlling transcriptional silencing, was reduced after adding SHH and FGF8. By attenuating this interaction, a group of genes can be upregulated (e.g., HDAC1). In addition, NCOR2 is involved in the Notch signaling pathway, which plays an important role in cell differentiation. Another group emerging in the network was claudin, including CLDN1 and CLDN3. Claudin is related to tight junctions and cell-to-cell adhesion [46]. Cell adhesion controls interactions of stem cells with their niche and signaling environment [47]. One member of the claudin group, CLDN6, was found to participate in the immunological elimination of hESCs and the prevention of cell outgrowth [48]. Hence, CLDN1 and CLDN3 may be involved in controlling the extension of hESCs and is worthy of further exploration.

In the IDN between LSB/S/F8/CHIR and LSB/S/F8 conditions (*D*_LSB/S/F8/CHIR-LSB/S/F8_; Figure 3(b)), there were four specific proteins with high relevance scores: GLRX, GTF2F2, FAM124B, and SEPT10. GLRX is differentially expressed in neuronal differentiation of human retinal pigment epithelial cells and in salamander spinal cord regeneration [49] and is involved in the estrogen pathway [50]. GTF2F2 encodes the general transcription factor IIF and is involved in NRF-1 regulated neurite growth [51] and the estrogen pathway [52]. FAM124B is a component of the CHD7- and CHD8-containing complex that is related to neurodevelopmental disorders [53]. Septin10 (SEPT10), an evolutionarily conserved group of GTP-binding and filament-forming proteins, interacts with components of cytoskeletons. Investigation of SEPT2, also a septin, has revealed a relationship between septin and neuronal development.

It was observed that some of these proteins (GLRX, GTF2F2, FAM124B, and SEPT10) showed more neuron-specific related functions after the addition of CHIR, while for some (NR2F2, NCOR2, CLDN1, and CLDN3), this was the case after adding SHH and FGF8. The latter group seems to be involved with more general processes of stem cell differentiation. The IDNs also provide a basis for further study. For example, the relations shown as thick edges in the IDNs (Figure 2) imply a greater amount of variation occurring during stage transitions, which may indicate essential roles. The interaction between METTL3 and METTL14 is one of the most varied, and its role in the directed differentiation of hESCs to DA neurons may be worth further investigation.

### 3.3. Relevance Scores of the Integrin, FGF, and WNT Functional Modules

The relevance scores of proteins in the previous section indicate the importance of essential candidates in the directed differentiation of hESCs to DA neurons. Complex biological phenomena are, however, the outcomes of intricate interactions among functional modules comprising several proteins with a specific function. Some of the significantly enriched GO terms are angiogenesis, the TGF-*β* signaling pathway, the PI3 kinase pathway, and the WNT signaling pathway, which is consistent with other enrichment analyses. For a clearer understanding of the mechanisms of the directed differentiation of hESCs to DA neurons, an interpretation from a functional module-level perspective is therefore indispensable. The relevance scores of proteins can be generalized to those of functional modules as shown in (8). The functional module-level interpretation of the directed differentiation can then be inferred from the scores. The prerequisite to calculating relevance scores of functional modules is to classify proteins into functional modules, which can be achieved through ontological or pathway analyses. Ontological analysis on the IDNs yielded several significant enriched functional modules, which are listed with their relevance scores in Table 2. Based on the ranking of relevance scores and *P* values, we focused on the integrin, FGF, and WNT functional modules in the following analysis.

**Figure 4:**
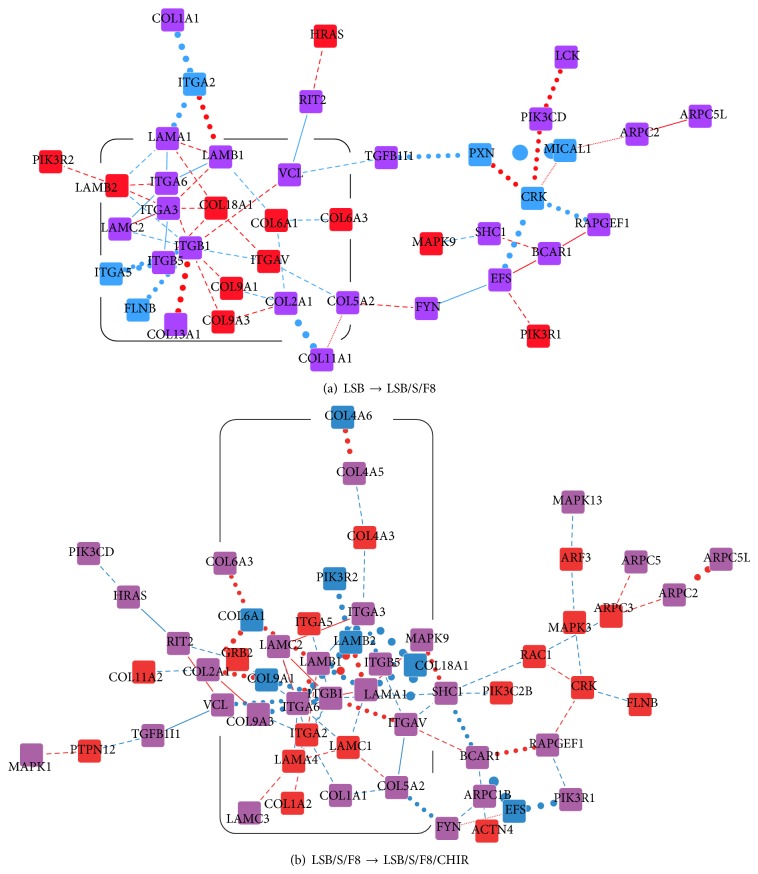
The interaction activity variations of the integrin functional module in the directed differentiation process at two differentiation stage transitions. (a) Interaction activity variations from the LSB stage to the LSB/S/F8 stage. (b) Interaction activity variations from the LSB/S/F8 stage to the LSB/S/F8/CHIR stage. Node and edge colors and edge line styles have the same interpretation as in Figure 2. The rounded squares are the core subnetworks in the integrin functional module.

The integrin functional module shows the highest rank by relevance score (Tables 2(a) and 2(b)), corresponding to both differentiation stage transitions from LSB to LSB/S/F8 and from LSB/S/F8 to LSB/S/F8/CHIR. In the differentiation stage transition from LSB to LSB/S/F8, the integrin functional module has a core subnetwork (the rounded square in Figures 4(a) and 4(b)) consisting of integrins (ITGA2, ITGA3, ITGA6, ITGAV, ITGB1, ITGB2, and ITGB5), collagens (COL2A1, COL5A2, COL6A1, COL9A1, COL9A3, COL11A1, and COL18A1), and laminins (LAMA1, LAMB1, LAMB2, and LAMC2). This core subnetwork links to VCL and FYN with enhancing interactions. VCL, a cytoskeletal protein, links to RAS (HRAS) and TGF*β* (TGFB1I1). TGFB1I1 then links to PXN, which is related to functions of focal adhesion, and LCK, a well-studied protein that can enhance differentiation of lymphoid cell lines by suppressing glucocorticoid sensitivity and apoptosis [54]. FYN, the other protein linking to the core network, controls cell growth. In addition, some actin-related proteins (ARPC2 and ARPC5L) are included in the core subnetwork through PXN. It is notable that many proteins in the integrin functional module also appear in pathways related to cancers. This explains the similarity between cancer cells and stem cells in their almost indefinite proliferation, immortality, and uncontrollable differentiation of induced stem cells. In the differentiation stage transition from LSB/S/F8 to LSB/S/F8/CHIR, the scale of the core network consisting of integrins, collagens, and laminins increased with the inclusion of further instances of these proteins (Figure 4(b)). The interactions from the core network to VCL and FYN diminished, implying that inhibition of apoptosis was not required. However, requirements for actin polymerization seemed to be increased. More actin-related proteins emerged in this IDN (Figure 4(b)). In addition, GRB2 emerged in the core network, acting as a suppressant of proliferative signals and triggering programmed cell death and being attenuated through interactions with RIT2 and ITGA6. Interactions related to cell growth and apoptosis inhibition in the previous stage transition (from LSB to LSB/S/F8) became more negative or vanished in the later stage transition (from LSB/S/F8 to LSB/S/F8/CHIR). In addition, actin polymerization and focal adhesion, which occur downstream of the integrin functional module, were more active in the later stage. These interaction variations indicate the necessity of regulation for cell survivability and neurogenesis.

**Figure 5:**
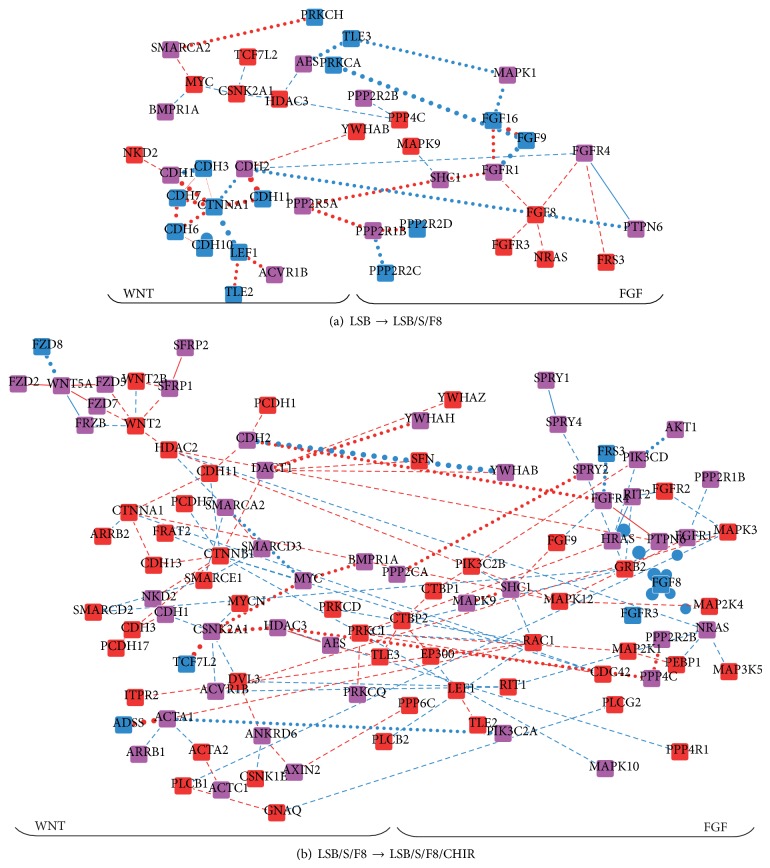
The interaction activity variations of the FGF and WNT functional modules in the directed differentiation process at two differentiation stage transitions. (a) Interaction activity variations from the LSB stage to the LSB/S/F8 stage. (b) Interaction activity variations from the LSB/S/F8 stage to the LSB/S/F8/CHIR stage. Node and edge colors and edge line styles have the same interpretation as in Figure 2.

The FGF functional module also had a high relevance score in the differentiation stage transitions (Tables 2(a) and 2(b)). As the WNT functional module has been implicated in the development of the thalamus [55], these two functional modules (FGF and WNT) were included in the following analysis. In the transition from LSB to LSB/S/F8, the interactions among cadherins (i.e., among CDH1, CDH2, CDH3, CDH7, CDH6, CDH10, and CDH11) were attenuated after adding SHH and FGF8. AES, a molecule in the WNT functional module, is related to neurogenesis. Its connection to FGF was attenuated and a negative interaction with HDAC3 emerged. CSNK2A1, which is related to axon guidance, had negative and attenuated interactions with its neighbor proteins (Figure 5(a)). In the transition from LSB/S/F8 to LSB/S/F8/CHIR (Figure 5(b)), FGF8 lost most of its interactions, and other FGFs and FGFRs connected to form a larger core network which included cadherins (e.g., CTNNB1, CDH11, CTNNA1, CDH13, PCDH7, and CDH3) and axon guidance molecules (CSNK2A1 and CSNK2A2). Some previously noted molecules (AES, CSNK2A1, and CSNK2A2) related to neurogenesis and axon guidance were reconnected to the network after adding CHIR. The reestablishment of connections between AES, CSNK2A1, and HDAC3 is required for the following differentiation process. Another important interaction occurred between WNT2 and HDAC2. Both of these proteins have been implicated in neuronal differentiation [56]. GRB2 also emerged in the core network and exerted its influence on cell growth in cooperation with the FGF and WNT functional modules.

The role of the FGF signaling pathway seemed to change between the early and later differentiation stage. During the first stage transition, the connections among the FGF, WNT, and integrin functional modules are built up for a general stem cell differentiation scheme. It may be an essential step to recruit them for further neuron differentiation, as the WNT and integrin functional modules are well-known for being involved in the neural development process. In the later stage transition, the FGF signaling pathway was involved in a more complex interaction network with neural differentiation-related proteins. The integrin signaling pathway also showed a similar trend; that is, many proteins with higher relevance scores in this pathway (e.g., LCK, FYN, and ARP) are involved in a general scheme of stem cell differentiation, while interactions with neurogenesis-related proteins appear later on. These proteins and their interactions may play essential roles in directed differentiation and require further investigation. Although the characteristics of the three discussed signaling pathways are familiar in the field of differentiation, dynamic PPI network-based relevance scores more explicitly indicate their roles in directed differentiation. The relevance scores indicate the diminishing demand for control of cell growth and apoptosis. The proposed relevance scores could therefore be a potential method to describe dynamic biological differentiation processes.

## 4. Conclusions

Identifying the keys in the directed differentiations of hESCs provides a basis to control the differentiation directions. The differentiation process can be treated as an evolution of interactions among proteins and functional modules in three differentiation stages. Hence, we built three PPI networks for the three differentiation stages corresponding to three different outcomes [5]. In general, a cell regulates itself through changing the interactions between molecules, for example, the inhibition and activation of proteins in PPIs, resulting in different responses. To investigate the variations of PPIs, we utilized IDNs to illustrate the interaction variations (enhanced or attenuated interaction) and the existence of proteins at two differentiation stages (diminished, emerged, or coexisting). Timely appearance and disappearance of molecules, interactions among molecules, and functional modules can promote the progress of the directed differentiation of hESCs into DA neurons. In this study, we used a computational method to investigate PPI network changes to dissect the directed differentiation process of hESCs into DA neurons and gain insight into the underlying mechanisms of directed differentiation.

Interaction activities between proteins have been identified by temporal gene expression profiles and then assembled into dynamic PPI networks for the three differentiation stages based on dynamic network modeling [9, 10, 22, 40]. Three dynamic PPI networks corresponding to three stages of hESC differentiation into DA neurons were constructed. A comparison of PPI networks at two differentiation stages revealed an interaction difference matrix depicted as an IDN (Figures 2–5) in which the interaction activity variations corresponding to the differentiation stage transition can be observed. Several known key proteins (OTX2, FOXA2, and LMX1A) in the directed differentiation of hESCs to DA neurons were inspected to justify the reliability of the resultant IDNs in the directed differentiation process. The variations of their interactions indicate the cellular functions promoting the progress of the directed differentiation. The relationships between these interaction variations and the cellular functions in the directed differentiation were verified by literature. To locate the essential proteins and functional modules in the directed differentiation, we furthermore quantified the importance of proteins and functional modules using relevance scores based on the dynamic PPI networks. According to the interaction activities identified by the dynamic network modeling, the variations in the interaction activity of a protein in the IDN can be calculated and defined as a relevance score. As a biological function consists of a series of protein-protein interactions, a larger variation of PPIs caused more profound effects on the differentiation outcomes. The relevance score is hence a representation of the significance of the protein in the differentiation stage transitions. Generalizing the concept from a protein to a cellular function, the significance of a functional module in the differentiation stage transitions can be also evaluated through relevance scores of functional modules.

As the differentiation stage transitions, the IDNs reflected the evolution of interaction variations. The high relevance scores in the proteins (e.g., MRPS34, EFEMP2, and STYXL1) and functional modules (e.g., LCK and FYN in the integrin signaling and subsiding interactions among cadherins in the FGF signaling) indicate that the evasion of apoptosis and enhanced survivability of differentiated stem cells may serve as general mechanisms of directed differentiation at the initial stage. In the transition from LSB/S/F8 to LSB/S/F8/CHIR, the proteins and functional modules with high relevance scores in the IDN are specific to neurogenesis and the functions of DA neurons (e.g., gonadotropin-releasing hormone secretion). The inhibition of apoptosis and the activation of cell growth declined after adding CHIR, a GSK3B inhibitor that also can activate WNT signaling. The activation of WNT signaling, although it is not a significant parameter in the enrichment analysis, was visible in the IDNs from LSB/S/F8 to LSB/S/F8/CHIR. In addition, cross-talk among the integrins, WNT, and FGF functional modules increased in the directed differentiation process (Figures 4 and 5) and was involved in determining the boundary of brain regions [55].

Several essential proteins and functional modules were described as underlying mechanisms of the directed differentiation process of hESCs into DA neurons using computational and literature verification. Many of these require further experiments to verify their ability to control the direction of differentiation. For now, a computational basis for understanding the directed differentiation process has been provided and may serve as a starting point for understanding the systematic mechanisms of directed differentiations as well as increasing the efficiency of stem cell-based therapies.

## Supplementary Material

The supplementary materials contain the whole interaction difference networks from LSB stage to LSB/S/F8 stage and from LSB/S/F8 stage to LSB/S/F8/CHIR stage (docx file) and the tables used in Cytoscape to visualize the interaction difference networks (xlsx files).

## Figures and Tables

**Table 1:** The ten proteins with the highest relevance scores.

Rank	LSB → LSB/S/F8		LSB/S/F8 → LSB/S/F8/CHIR	
	Relevance score	Protein	Relevance score	Protein
1	33.71857	METTL14	33.71857	METTL14
2	15.33615	METTL3	16.94178	METTL3
3	14.93786	NR2F2	9.465864	GLRX
4	4.348328	POLR2I	3.366032	POLR2I
5	2.916923	CLDN3	2.441295	EFEMP2
6	2.682951	MRPS34	2.041421	MRPS34
7	2.682884	NCOR2	2.001651	GTF2F2
8	2.457388	EFEMP2	1.884138	FAM124B
9	2.227754	CLDN1	1.859985	STYXL1
10	2.141034	STYXL1	1.42109	SEPT10

**(a):** 

Rank	LSB → LSB/S/F8		
	Relevance score	Function	*P* value of ontology analysis
1	7.627697307	Huntington disease	5.55*E* − 03
2	5.389941296	Integrin signaling pathway	1.35*E* − 07
3	4.865942264	Gonadotropin releasing hormone receptor pathway	1.87*E* − 02
4	4.776453757	Angiogenesis	2.76*E* − 10
5	3.318549657	PDGF signaling pathway	1.10*E* − 02
6	3.13212684	Cytoskeletal regulation by Rho GTPase	2.68*E* − 04
7	3.120593534	FGF signaling pathway	1.69*E* − 03
8	3.016342937	EGF receptor signaling pathway	8.17*E* − 05
9	2.417680991	TGF-beta signaling pathway	9.86*E* − 03
10	2.36486466	Parkinson disease	2.78*E* − 02

**(b):** 

Rank	LSB/S/F8 → LSB/S/F8/CHIR		
	Relevance score	Function	*P* value of ontology analysis
1	7.085580216	Gonadotropin releasing hormone receptor pathway	2.84*E* − 04
2	6.595569241	Integrin signaling pathway	3.54*E* − 08
3	4.956190934	FGF signaling pathway	1.68*E* − 06
4	4.634381934	Angiogenesis	6.45*E* − 06
5	4.535296087	EGF receptor signaling pathway	2.35*E* − 06
6	3.702903524	Huntington disease	9.60*E* − 03
7	3.299706184	PDGF signaling pathway	2.75*E* − 02
8	2.664383051	Parkinson disease	8.74*E* − 05
9	2.640768983	TGF-beta signaling pathway	3.05*E* − 02
10	2.61175047	RAS pathway	1.18*E* − 03
