# Supplementary material for: Dynamic Network-Based Relevance Score Reveals Essential Proteins and Functional Modules in Directed Differentiation
Source: Stem Cells Int. 2015 Apr 21;2015:792843. doi: 10.1155/2015/792843 (PMC4419265; doi:10.1155/2015/792843)
Supplement: Supplementary file 1 — The supplementary materials contain the whole interaction difference networks from LSB stage to LSB/S/F8 stage and from LSB/S/F8 stage to LSB/S/F8/CHIR stage (docx file) and the tables used in Cytoscape to visualize the interaction difference networks (xlsx files). [file 792843.f1.zip › New folder/mat.792843.v1 (2).docx]

Figure S1A. The whole interaction difference network from LSB stage to LSB/S/F8 stage.

The blue node indicates the node exists only in the dynamic PPI network of LSB stage. The red node indicates the node exists only in the dynamic PPI network of LSB/S/F8 stage. The purple node indicates the node exists in both the dynamic PPI network of LSB and LSB/S/F8 stage.

Figure S1B. The whole interaction difference network from LSB/S/F8 stage to LSB/S/F8/CHIR stage.

The blue node indicates the node exists only in the dynamic PPI network of LSB/S/F8 stage. The red node indicates the node exists only in the dynamic PPI network of LSB/S/F8/CHIR stage. The purple node indicates the node exists in both the dynamic PPI network of LSB/S/F8 and LSB/S/F8/CHIR stage.
